# Evaluation of the bioresistance profile of enterobacteria isolated from faeces of children with diarrhoea in the town of Koula-Moutou, Gabon: prospective study

**DOI:** 10.11604/pamj.2022.43.63.25276

**Published:** 2022-10-07

**Authors:** Rolande Mabika Mabika, Sandrine Lydie Oyegue Liabagui, Franck Mounioko, Alain Souza, Jean Fabrice Yala

**Affiliations:** 1 Laboratory of Molecular and Cellular Biology, Microbiology Team, Agrobiology Research Unit, Masuku University of Science and Technology, BP 067 Franceville, Gabon,; 2 Laboratory of Research in Immunology, Parasitology and Microbiology, Regional Doctoral School of Central Africa in Tropical Infectiology, Masuku University of Science and Technology, Franceville, Gabon,; 3 Vector Ecology Laboratory, Tropical Ecology Research Institute, Libreville, Gabon

**Keywords:** Panresistance, multiresistance, diarrhoea, enterobacteria, Gabon

## Abstract

**Introduction:**

the emergence and expansion of multidrug resistance in Enterobacteriaceae responsible for various infections are increasing in the world. This study was designed to determine the phenotypic profiles of the resistance of enterobacteria strains isolated from the faeces of children with diarrhoeal diseases and to classify them according to the type of resistance.

**Methods:**

screening was carried out on 98 isolates divided into 2 groups: opportunistic pathogens and strict enteropathogens. Their sensitivity to 13 antibiotics was evaluated by the Mueller Hinton agar medium diffusion method.

**Results:**

a strong resistance to different classes of β-lactams was found in the strains, 74.0% (n=45) and 83.3% (n=31) for opportunists and enteropathogens, respectively. These strains were completely resistant to doxycycline and erythromycin (100%; n=98) for both types of bacteria. Opportunists and enteropathogens were 95.1% (n=58) and 94.6% (n=35) resistant to gentamicin, and 31.1% (n=19) and 35.1% (n=13) resistant to chloramphenicol, respectively. Similarly, the total resistance of strains was observed with ofloxacin and amounted to 98.4% (n=60) and 96.7% with levofloxacin and norfloxacin, respectively. The analysis of β-lactam resistance phenotypes revealed a dominance of the carbapenemase-producing strains (28.6%; n=28). However, 24.3% (n=9) of enteropathogens were pan-resistant versus 19.7% (n=12) for opportunists.

**Conclusion:**

the results of this study indicate a worryingly high level of antibiotic resistance in enterobacteria which might tend towards total resistance.

## Introduction

Diarrhoeal diseases due to their endemic nature, represent a public health problem in developing countries whose annual global incidence is estimated at 1.7 billion cases in children aged less than 5 years old [1,2]. In the majority of diarrhoeal cases of infectious origin, a variety of viral and bacterial pathogens are responsible and are most commonly found in children aged 0 to 5 years old [3,4]. The management of diarrhoeal diseases in African sub-Saharan countries is in most cases based on a probabilistic treatment without a prior research of the responsible agent mostly due to the absence or the obsolescence of the technical platforms of health structures [2,5]. This approach, to a lesser extent combined with the misuse of antibiotic prescriptions in some cases, is a risk factor for modifying the ecology of bacteria mainly in bacterial pathogens [6,7].

Enterobacteria, which are the most common causative agents of human infections, currently show a high antibiotic resistance panel due to the acquisition of resistance genes by this family [8,9]. Diarrhoeagenic *Escherichia coli, Salmonella* spp, *Shigella* spp and *Yersinia* spp are the major *Enterobacteriaceae* involved in diarrhoeal diseases and in recent years, an increase in antibiotic resistance used in treatment has been observed with the emergence of broad-spectrum β-lactamase-producing strains [10,11]. Furthermore, in the Sahel region, the emergence of strains resistant to amoxicillin, chloramphenicol, co-trimoxazole but also to cephalosporins and fluoroquinolones has been reported in the literature [12]. Moreover, with the release of enterobacteria from the producers of carbapenemases, the therapeutic capital is largely compromised [9,13-15]. This expansion and spread of enterobacteria resistance to almost all classes of antibiotics is currently a major problem in the management of these diseases in developing countries [15-17].

As of now, only few studies have investigated the molecular mechanisms of antimicrobial resistance among isolates isolated from patients in the sub-Saharan African region, particularly diarrhoeal patients, mainly due to the limited number of adequate infrastructures and researchers available on the continent [18]. Given this situation, it is very important to set up surveillance networks to gain information on the spread of ESBLs and other resistance phenotypes in diarrhoeal pathogens to implement good clinical management during treatments.

It is in this context that this study aims to determine the antibiotic susceptibility profile of germs isolated from diarrhoeal faeces in children aged 0 to 5 years old in the town of Koula-Moutou and establish the phenotypes of resistance of circulating enterobacteria in it.

## Methods

**Study design and setting:** all enterobacteria strain isolated from diarrhoeal faeces of children aged 0 to 5 years old hospitalized or received as outpatients were included in this study [19]. A total of 98 isolates from 11 genera were screened to assess their susceptibility to antibiotics. These strains were separated into two groups: 37 strict enteropathogens including *Salmonella enterica, Salmonella Paratyphi A, Salmonella spp., Salmonella Typhi, Shigella sonnei, Shigella spp and Yersinia spp.; and 61 opportunistic enteropathogens (Citrobacter braakii, freundii and koseri, Escherichia vulneris, Enterobacter aerogenes and cloacae, Klebsiella oxytoca and pneumoniae, Kluyvera spp, Pantoea spp, Raoultella ornithinolytica and terrigena)*.

**Laboratory analysis:** thirteen molecules belonging to five families of antibiotics (Bio-Rad, Marnes-la-Coquette, France and BBLTM Sensi-Disc) were used: for β-lactam, 3 penicillins including amoxicillin (AMX, 25 µg) (aminopenicillins), amoxicillin + clavulanic acid (AMC, 20/10 µg) and piperacillin + tazobactam (TZP, 100/10 µg); 2 cephalosporins including one of 3rd and one of 4th generation: ceftazidime (CAZ, 30µg), cefepime (FEP, 30µg) and a carbapenem, imipenem (IPM, 10µg). For families of aminoglycosides, macrolides, phenicolates and tetracyclines, the molecules were gentamicin (GME, 30 µg), erythromycin (ERY, 15 µg), chloramphenicol (CHL, 30 µg) and doxycycline (DOX, 30 µg), respectively. For the fluoroquinolone family, 3 molecules were tested: levofloxacin (LVX, 5µg), norfloxacin (NXN, 10µg) and ofloxacin (OFX, 5µg). These have not been tested on the different strict enteropathogenic strains in this study. The study of antibiotic susceptibility was carried out using the technique of diffusion of antibiotic discs in Mueller Hinton agar medium according to the Kirby-Bauer technique. Standard 0.5 McFarland inoculi was prepared in sterile physiological saline (0.9% NaCl) of 18-24 hours pure colonies. The interpretation of the results of the activity was based on the standards of the Antibiogram Committee of the French Society (CASFM) recommendations 2019 v.2.0, except for erythromycin and doxycycline whose standards are in force and those of the CASFM 2013 and 2015, respectively. The identification of natural resistance phenotypes as well as the main mechanisms of acquired resistance to β-lactams from diarrhoeal fecal isolates, as well as the phenotypic classification of the different types of carbapenemases was performed by the interpretation of the antibiogram at specific ATB [20]. The strains were categorized as multidrug-resistant bacteria (MRB) for all those that had resistance to at least 3 antibiotic molecules belonging to the same family or different families [21]. Moreover, all those who presented a phenotype “resistant” to all 13 antibiotic molecules considered in this study were categorized as pan-resistant bacteria (PRB).

**Statistical analysis:** the chi-squared test was used to compare the different proportions of resistance with a threshold of significance set at 5%. “Intermediate” results were included in the “resistant” category. This test was performed using the R software v3.2.2.

## Results

The screening of the strains with molecules belonging to the different classes of the β-lactam family and to the 4 antibiotic molecules belonging to the aminoglycoside, phenicolated, macrolide and tetracycline families was done on the two categories of isolated strains. Their phenotypic results are recorded in Table 1. The analysis of the results shows a strong 95.9% (n=94) resistance to aminopenicillins (AMX) whose proportions are more or less similar to the two categories of strains with 96.7% (n=59) for opportunistic pathogens and 94.9% (n=35) for strict enteropathogens, respectively. In addition, the chi-squared test shows that there is a significant difference in behaviour depending on the bacterial groups (p < 0.0005). These percentages are slightly lower when these molecules are conjugated with the β-lactamase inhibitors: AMC (89.8%, n=88) and TZP (70.4%; n=69). Regarding the cephalosporin class, 91.8% (n=90) and 81.6% (n=80) of ceftazidime and cefepime, respectively, have high resistance levels. The opportunistic pathogens have resistance rates of 88.5% (n=54) and 70.5% (n=43), respectively, for ceftazidime and cefepime whereas those of enteropathogens are 97.3% (n=36) and 100% (n=37) for the same molecules. Also, a significant difference was recorded between these two groups for cefepime (p = 0.027). Finally, carbapenems remain the most active β-lactams with an average overall resistance rate of 36.7% (n=36), with the prevalences of 29.5% (n=18) and 48.6% (n=18) for opportunistic pathogens and strict enteropathogens (p <0.0005), respectively.

**Table 1:** resistance profiles of enterobacteria to β-lactams and 4 antibiotics belonging to various families

Names of bacterial strains	N	AMX	AMC	TZP	CAZ	FEP	IMP	GME	CHL	ERY	DOX
		n (% I/R)	n (% I/R)	N (% I/R)	n (% I/R)	n (% I/R)	n (% I/R)	n (% I/R)	n (% I/R)	n (% I/R)	n (% I/R)
**Opportunistic pathogens**											
Citrobacter braakii	**1**	1 (100.0)	0 (0.0)	0 (0.0)	0 (0.0)	0 (0.0)	0 (0.0)	1 (100.0)	0 (0.0)	1 (100.0)	1 (100.0)
Citrobacter freundii	**1**	1 (100.0)	1 (100.0)	1 (100.0)	1 (100.0)	0 (0.0)	0 (0.0)	1 (100.0)	1 (100.0)	1 (100.0)	1 (100.0
Citrobacter koseri	**1**	1(100.0)	1 (100.0)	1 (100.0)	1 (100.0)	1 (100.0)	1 (100.0)	1 (100.0)	0 (0.0)	1 (100.0)	1 (100.0)
Enterobacter aerogenes	**5**	5 (100.0)	5 (100.0)	4 (80.0)	4 (80.0)	2 (40.0)	2 (40.0)	5 (100.0)	3 (60.0)	5 (100.0)	5 (100.0)
Enterobacter cloacae	**2**	2 (100.0)	2 (100.0)	2 (100.0)	2 (100.0)	2 (100.0)	0 (0.0)	2 (100.0)	1 (50.0)	2 (100.0)	2 (100.0)
Escherichia vulneris	**2**	2 (100.0)	2 (100.0)	2 (100.0)	2 (100.0)	2 (100.0)	0 (0.0)	2 (100.0)	2 (100.0)	2 (100.0)	2 (100.0)
Klebsiella oxytoca	**3**	3 (100.0)	3 (100.0)	2 (66.7)	3 (100.0	3 (100.0)	1 (33.3)	2 (66.7)	1 (33.3)	3 (100.0)	3 (100.0)
Klebsiella pneumoniae	**4**	3 (75.0)	3 (75.0)	3 (75.0)	4 (100.0)	4 (100.0)	3 (75.0)	4 (100.0)	1 (25.0)	4 (100.0)	4 (100.0)
Kluyvera spp	**5**	5 (100.0)	5 (100.0)	3 (60.0)	5 (100.0)	5 (100.0)	1 (20.0)	5 (100.0)	1 (20.0)	5 (100.0)	5 (100.0)
Pantoea spp	**1**	1 (100.0)	1 (100.0)	0 (0.0)	0 (0.0)	1 (100.0)	0 (0.0)	1 (100.0)	0 (0.0)	1 (100.0)	1 (100.0)
Raoultella ornithinolytica	**21**	21 (100.0)	17 (81.0)	16 (76.2)	21 (100.0)	15 (71.4)	5 (23.8)	19 (90.5)	4 (19.0)	21 (100.0)	21 (100.0)
Raoultella terrigina	**4**	4 (100.0)	4 (100.0)	2 (50.0)	4 (100.0)	4 (100.0)	2 (50.0)	4 (100.0)	0 (0.0)	4 (100.0)	4 (100.0)
Serratia fonticola	**5**	5 (100.0)	4 (80.0)	4 (80.0)	2 (40.0)	2 (40.0)	0 (0.0)	5 (100.0)	1 (20.0)	5 (100.0)	5 (100.0)
Serratia liquefaciens	**2**	2 (100.0)	2 (100.0)	1 (50.0)	2 (100.0)	0 (0.0)	1 (50.0)	2 (100.0)	2 (100.0)	2 (100.0)	2 (100.0)
Serratia odorifera 1	**4**	3 (75.0)	4 (100.0)	3 (75.0)	3 (75.0)	2 (50.0)	1 (25.0)	4 (100.0)	2 (50.0)	4 (100.0)	4 (100.0)
Total	**61**	**59 (96.7)**	**54 (88.5)**	**44 (72.1)**	**54 (88.5)**	**43 (70.5)**	**17 (27.9)**	**58 (95.1)**	**19 (31.1)**	**61 (100.0)**	**61 (100.0)**
**Enteric pathogens**											
Salmonella enterica	**6**	6 100.0)	6 (100.0)	3 (50.0)	6 (100.0)	6 (100.0)	4 (66.7)	6 (100.0)	2 (33.3)	6 (100.0)	6 (100.0)
Salmonella Paratyphi A	**3**	3 (100.0)	3 (100.0)	3 (100.0)	3 (100.0)	3 (100.0)	0 (0.0)	3 (100.0)	1 (33.3)	3 (100.0)	3 (100.0)
Salmonella spp	**17**	15 (88.2)	15 (88.2)	11 (64.7)	17 (100.0)	17 (100.0)	10 (58.8)	16 (94.1)	5 (29.4)	17 (100.0)	17 (100.0)
Salmonella Typhi	**4**	4 (100.0)	3 (75.0)	3 (75.0)	3 (75.0)	4 (100.0)	3(75.0)	4 (100.0)	1 (25.0)	4 (100.0)	4 (100.0)
Shigella sonnei	**2**	2 (100.0)	2 (100.0)	2 (100.0)	2 (100.0)	2 (100.0)	0 (0.0)	2 (100.0)	0 (0.0)	2 (100.0)	2 (100.0)
Shigella spp	**3**	3 (100.0)	3 (100.0)	3 (100.0)	3 (100.0)	3 (100.0)	1 (33.3)	2 (66.7)	2 (66.7)	3 (100.0)	3 (100.0)
Yersinia pestis	**2**	2 (100.0)	2 (100.0)	0 (0.0)	2 (100.0)	2 (100.0)	0 (0.0)	2 (100.0)	2 (100.0)	2 (100.0)	2 (100.0)
Total	**37**	**35 (94.6)**	**34 (91.9)**	**25 (67.6)**	**36 (97.3)**	**37 (100.0)**	**18 (48.6)**	**35 (94.6)**	**13 (35.1)**	**37 (100.0)**	**37 (100.0)**
**Global Percentage of resistance**	**98**	**94 (95.9)**	**88 (89.8)**	**69 (70.4)**	**90 (91.8)**	**80 (81.6)**	**36 (36.7)**	**93 (94.9)**	**32 (32.7)**	**98 (100.0)**	**98 (100.0)**

**AMX:** amoxicillin; **AMC:** amoxicillin + clavulanic acid; **TZP:** piperacillin / tazobactam; **CAZ:** ceftazidime; **FEP:** cefepime; **IMP:** imipenem ; **GME** : gentamicine ; **CHL** : chloramphénicol ; **ERY** : érythromycine ; **DOX** : doxycycline.

These results show that the different isolates, both opportunistic pathogens and enteropathogens, have a total resistance to erythromycin (100%; n=98) and doxycycline (100%; n=98) without any significant difference between the two groups (p = 0.08). In addition, a strong resistance to gentamicin is also observed (94.9%; n=93). For chloramphenicol, an average resistance rate of 32.7% (n=32) was recorded with resistance rates of 31.1% (n=19) and 35.1% (n=13) for opportunistic pathogens and strict enteropathogens (p = 0.98), respectively (Table 1). The susceptibility of 61 isolates of opportunistic pathogens was assessed against 3 molecules of antibiotics belonging to the fluoroquinolone family (Table 2). A very high resistance is observed in opportunistic pathogens, generally without any significant difference with respect to the 3 molecules (p = 0.66). A 100% (n=61) resistance rate is reported for ofloxacin, 98.4% (n=60) for levofloxacin and 96.7% (n=59) for norfloxacin.

**Table 2:** resistance profile of enterobacteria isolated from quinolone diarrhoea

Names of bacterial strains	N	LVX	OFX	NXN
		n (% I/R)	n (% I/R)	n (% I/R)
**Opportunistic pathogens**				
Citrobacter braakii	**1**	1(100,0)	1 (100,0)	1 (100,0)
Citrobacter freundii	**1**	1 (100,0)	1 (100,0)	1 (100,0)
Citrobacter koseri	**1**	1 (100,0)	1 100,0)	1 (100,0)
Enterobacter aerogenes	**5**	5 (100,0)	5 (100,0)	5 (100,0
Enterobacter cloacae	**2**	2 (100,0)	2 (100,0)	2 (100,0)
Escherichia vulneris	**2**	2 (100,0)	2 (100,0)	2 (100,0)
Klebsiella oxytoca	**3**	2 (66,7)	3 (100,0)	2 (66,7)
Klebsiella pneumoniae	**4**	4 (100,0)	4 (100,0)	4 (100,0)
Kluyvera spp	**5**	5 (100,0)	5 (100,0)	5 (100,0)
Pantoea spp	**1**	1 (100,0)	1 (100,0)	1 (100,0)
Raoultella ornithinolytica	**21**	21 (100,0)	21 (100,0)	20 (95,2)
Raoultella terrigina	**4**	4 (100,0)	4 (100,0)	4 (100,0)
Serratia fonticola	**5**	5 (100,0)	5 (100,0	5 (100,0
Serratia liquefaciens	**2**	2 (100,0)	2 (100,0)	2 (100,0)
Serratia odorifera 1	**4**	4 (100,0)	4 (100,0)	4 (100,0)
**Global Percentage of resistance**	**61**	**60 (98.4)**	**61 (100.0)**	**59 (96.7)**

**LVX**: levofloxacin; **OFX**: ofloxacin; **NXN**: norfloxacin

### Mechanisms of phenotypic resistance by the production of β-lactamase enzymes

The Carbase resistance mechanism is the most common phenotype in enterobacterial strains, with a prevalence of 28.6% (n=28) (Table 3). The resistance rates are 21.3% (n=13) and 40.5% (n=15) for the opportunistic pathogen and strict enteropathogens groups, respectively. Moreover, class A/B is the most representative among the phenotypic classification of Carbase types with a frequency of 89.3% (n=87), of which 100.0% (n=61) and 80.0% (n=29) are found in the opportunists and in the strict enteropathogens, respectively. In contrast, Class D Carbase-positive strains have a low prevalence (10.7%; n=10). This phenotype is followed by the one associated with the combination of the production of an ESBL and/or a Case HP whose prevalence is 23.5% (n=23) and with 16.4% (n=10) of cases observed in opportunistic pathogens versus 35.1% (n=13) in strict enteropathogens. Strains of enterobacteria resistant to antibiotics by production Case BN have a prevalence of 20.4% (n=20) while those, a prevalence of 19.4% (n=19) is recorded for enterobacterial strains producing ESBL.

**Table 3:** phenotype of resistance of enteric strains to β-lactams

Names of bacterial strains		Pase	BLSE	Case BN	BLSE and/or CaseHP	Carbase
	N	n (%)	n (%)	n (%)	n (%)	n (%)
**Opportunistic pathogens**						
Citrobacter braakii	**1**	-	-	1 (100.0)	-	-
Citrobacter freundii	**1**	-	-	1 (100.0)	-	-
Citrobacter koseri	**1**	-	-	-	-	1(100.0)
Enterobacter aerogenes	**5**	-	-	3 (40.0)	-	2 (60.0)
Enterobacter cloacae	**2**	-	-	2 (100.0)	-	
Escherichia vulneris	**2**	-	1 (50.0)	-	1 (50.0)	-
Klebsiella oxytoca	**3**	-	1 (33.3)	-	2 (66.7)	-
Klebsiella pneumoniae	**4**	1 (25.0)	1 (25.0)	-	-	2 (50.0)
Kluyvera spp	**5**	-	4 (80.0)	-	-	1 (20.0))
Pantoea spp	**1**	1 (100.0)	-	-	-	-
Raoultella ornithinolytica	**21**	3 (14.3)	4 (19.0)	5 (23.8)	6 (28.6)	3 (14.3)
Raoultella terrigina	**4**	-	2 (50.0)	-	-	2 (50.0)
Serratia fonticola	**5**	-	1 (20.0)	3 (60.0)	1 (20.0)	-
Serratia liquefaciens	**2**	-	-	1 (50.0)	-	1 (50.0)
Serratia odorifera 1	4	1 (25.0)	-	2 (50.0)	-	1 (25.0)
Total	61	6 (9.8)	14 (23.0)	18 (29.5)	10 (16.4)	13 (21.3)
**Enteropathogens**						
Salmonella enterica	**6**	-	-	-	2 (33.3)	4 (66.7)
Salmonella Paratyphi A	**3**	-	-	-	3 (100.0)	-
Salmonella spp	**17**	2 (11.8)	3 (17.6)	1 (5.9)	3 (17.6)	8 (47.1)
Salmonella Typhi	**4**	-	-	1 (25.0)	-	3 (75.0)
Shigella sonnei	**2**	-	2 (100.0)	-	-	-
Shigella spp	**3**	-	-	-	3 (100.0)	-
Yersinia pestis	**2**	-	-	-	2 (100.0)	-
Total	**37**	**2 (5.4)**	**5 (13.5)**	**2 (5.4)**	**13 (35.1)**	**15 (40.5)**
**Global Percentage of resistance**	**98**	**8 (8.2)**	**19 (19.4)**	**20 (20.4)**	**23 (23.5)**	**28 (28.6)**

**Pase:** broad spectrum penicillinases; **Case BN**: low level cephalosporinase; **Case HP:** cephalosporinase High level; **ESBL:** broad-spectrum β-lactamases; **Carbase:** carbapenemases

### Characterization of the type of resistance

The results reveal that globally 78.6% (n=77) of the isolated enterobacteria strains are MRB and 21.4% (n=21) are PRB. Regarding opportunistic pathogens, only the *Citrobacter braakii, Citrobacter koseri, Enterobacter cloacae, Escherichia vulneris, Pantoea spp, Raoultella terrigina* and *Serratia fonticola* species have a MRB profile, which represents 7 species out of 15 for this group. As for the group of strict enteropathogens, 3 species are only MRB: *Salmonella Paratyphi A, Shigella sonnei* and *Yersinia pestis*.

## Discussion

The objective of this study was to establish the antibiotic susceptibility profile of opportunistic *Enterobacteriaceae* and strict pathogens associated with diarrhoea cases in children in the city of Koula-Moutou in Gabon. Enterobacteria isolated from diarrhoeal faeces have high levels of resistance to the different families (β-lactam, quinolones) of tested antibiotics, among which are molecules prescribed by health personnel in case of suspicion of infectious diarrhoea. However, for the molecules of the last therapeutic lines (carbapenems) and also for chloramphenicol, low rates of resistance are recorded suggesting in particular their weak utilization or the installation of the mechanisms of resistance. The analysis of β-lactam resistance phenotypes revealed a dominance of the carbapenemase-producing strains and those producing the ESBL/Case. According to the different bacterial groups, the strains present have more of a multiresistant bacterial character despite the low rate of the so-called pan-resistant bacteria recorded.

The resistance profiles recorded in this study corroborate those of the many studies concerning enterobacteria that are involved in many human infections [22,23]. The high levels of β-lactam resistance recorded in this work are similar to those reported by other studies [24,25]. Indeed, for opportunistic pathogens, AMX resistance levels belonging to the aminopenicillin class with ampicillin are very high. These results are similar to those recorded against AMP in the studies of Azimi *et al*. in Teheran and Monira *et al*. in Bangladesh [25,26]. Similarly, the high AMC resistance rates obtained in this study are comparable to those of the study by Azimi *et al*. [25]. Compared to enteropathogens, *Salmonella* isolates also show high rates of resistance to amoxicillin alone or with added clavulanic acid. These rates are comparable to those reported by Randrianirina *et al*. (35.7%) in Madagascar [24]. Furthermore, resistance prevalences similar to other studies were recorded for strains of *Shigella* spp, for the aminopenicillin class [27]. These high levels of resistance to aminopenicillins, in particular AMC, could be explained by current practices in the hospital environment because this molecule is more commonly prescribed in the treatment of various conditions [2,22].

Regarding the cephalosporins, the resistance rates found in this study are significantly higher than those of other studies for both opportunistic *Enterobacteriaceae* and enteropathogens [12,24,26]. In developing countries, the use of antibiotics for prophylaxis and therapy is widespread, particularly in the treatment of infantile bacterial diarrhoeal diseases in addition to urinary tract infections [2,22]. These practices would contribute to the selection of chromosomal and plasmidic hyperproductive mutants of cephalosporinases during treatment [9,28]. Indeed, this phenomenon is particularly found in *Enterobacter cloacae, aerogenes, Citrobacter freundii, Klebsiella spp* and *Salmonella spp* [9] species as is the existence of the phenomenon of cross-transmission of resistance genes. This is particularly observed in enterobacteria and can explain the high levels of resistance in this study. In addition, the global ESBL, which are third-generation β-lactamases that hydrolyze cephalosporins and aztreonam, is responsible for increasing the resistance of these strains to the majority of β-lactams [29,30] including cephalosporins.

Regarding the class of penems, an overall resistance of 36.7% against IMP is obtained, with a rate of 27.9% for opportunists. This prevalence is similar to the one obtained by Azimi *et al*. Their work has indeed recorded resistance rates of 18.5% and 53.6% for *Klebsiella spp* and *Citrobacter spp*, respectively, comparable to those of our study estimated at 16.5% for *Klebsiella spp* and 33.3% for *Citrobacter spp*. In Niger and Morocco, isolated enteropathogens had low resistance rates to IMP of 0%, 0%, and 0%; and 0%, 0% and 1.4% for *Salmonella spp, Shigella spp* and diarrhoeagenic *Escherichia coli* strains [12,21], respectively. In our study these resistance rates are 50.1% and 16.7% for *Salmonella spp* and *Shigella*, respectively. These obtained prevalences could be the result of the dissemination of carbapenemase-producing strains which are described as epidemiogenic [13,31]. Or in this case, the association of a decrease in porin (alteration of membrane permeability) with the production of an ESBL or Case [14,29] could possibly be one of the mechanisms developed by these isolates which would explain the prevalence of resistance to this antibiotic.

The strong resistance to quinolones in this study highlights the spread of resistant enterobacteria to this antibiotic family, particularly in *E. coli, Salmonella spp* and *Shigella spp* [11,32,33]. Moreover, the prophylactic treatments have been refocused on β-lactams and fluoroquinolones for bacterial diarrhoeal pathologies [34], this therapeutic alternative could have contributed to the expansion of multiresistant strains against these molecules in enterobacteria. Based on these results, it appears that the spectrum of active molecules against enterobacteria in the town of Koula-Moutou is very limited and raises a real public health problem in the management of diarrhoeal patients. The different resistance profiles to the different families of antibiotics recorded in this study corroborate the expansion of the resistance phenomenon of enterobacteria, which is mainly due to the production of Î^2^-lactamase enzymes, in particular the production of penicillinases, broad-spectrum β-lactamases (ESBL), chromosomal or plasmidic cephalosporinases (Case) and carbapenemases [30,35]. Obviously, to survive drug pressure due to antibiotics, bacteria have developed survival strategies by diversifying their acquired resistance mechanisms, mainly through the acquisition of mobile genetic elements [9,26], in addition to regulatory systems that can sometimes control the expression of natural β-lactamases [20]. Thus, 23.5% of the strains studied produce an ESBL associated with the production of a Case, whose prevalence is higher than that obtained in Algeria (14.3%) [6] and in the Netherlands (4.5%) [36]. In addition, 19.4% of isolated enterobacteria are ESBL producers, with a high frequency compared to that found by Chervet *et al*. in Paris (4%) [23], lower than that obtained by Yala *et al*. in Gabon (51%) [37] and on the African continent (77.8%) [38]. In this study, 20.4% of isolates produce Case, which would increase the resistance potential of these isolates, undoubtedly explaining the high levels of resistance observed in this study. Their isolation frequency is higher than that obtained by van Den Bunt *et al*. in the Netherlands, which was 0.2% for children under 5 years old and 0.4% for the overall population [39]. Furthermore, 28.6% of *Enterobacteriaceae* produce carbapenemases in this study, whose prevalence is lower than that obtained by Mabika Mabika *et al*. (2019) [40]. Ultimately, the mechanism of enzyme production in antibiotic resistance is widespread and diversified within the enterobacterial strains studied.

At present, the global epidemiological context is marked by the emergence of MRB dominated by Gram-negative bacteria whose main representatives are *Enterobacteriacea, Pseudomonas aeruginosa* and *Acinetobacter* which are resistant to large classes of antibiotics, sometimes cross-referenced [41,42]. The results of this study corroborate in particular the observations reported in the literature. Indeed, an overall prevalence of MRB enterobacteria isolates estimated at 78.6% is recorded with a frequency higher than that obtained in Morocco (47.7%) [21]. This high prevalence of MRB could be explained and confirmed by the production of β-lactamases of the ESBL, Case and carbapenemase type by the isolates which give them resistance to β-lactams and to the families of antibiotics tested as well [41]. Genes coding for these enzymes are usually located on plasmids often associated with other resistance genes to other classes of antibiotics [9], as well as other mobile genetic elements (transposons, class 1 and 2 integrations) [26]. Consequently, the genetic multiplicity of resistance on these different elements, and the rapid adaptability could contribute to the emergence of mutants resistant to all the usual classes of antibiotics, possibly explaining the 21.4% of the pan-resistant isolates obtained in this study.

Moreover, it has been reported in the literature that commensal strains may constitute reservoirs of antibiotic resistance in the community [26]. Although the mechanism of resistance was identified only phenotypically in this study, for the 19.7% of isolated PRB opportunists, a frequency of 21.3% of EPC is identified, arguably in favour of BHRe cases. Our prospective study clearly established the emergence of diarrhoeal bacterial strains in several families of first-line antibiotics phenotypically. However, one of the main limitations of this study is the lack of molecular characterization of resistance genes on our strains that would have better established the risk of dissemination in our country.

## Conclusion

This study highlights the very high prevalence of *Enterobacteriaceae* isolated from diarrhoeal faeces against antibiotics commonly used in the treatment of diarrhoeal diseases in Gabon. Although the majority of these strains are multiresistant, carbapenems and chloramphenicol still remain the most active molecules in most of them despite the resistance observed in rare cases. Furthermore, this study also reveals a low average proportion of carbapenem-resistant strains, although the combination of ESBL with a cephalosporinase was the most common phenotype in this study. Specific tests should be routinely performed to better characterize these β-lactamase resistance phenotypes, particularly regarding carbapenem resistance.

### What is known about this topic

- The emergence of antibiotic-resistant strains in diarrhoeal isolates;
- The risk of increasing bacterial resistance through the practice of using empirical treatments.

### What this study adds

- The establishment of susceptibility profiles of diarrhoeal isolates in a town in Gabon;
- Profiles of resistance mechanisms circulating in the said city and by extension at the national level.
